# Preparation of Ti_3_C_2_T_x_ modified rare earth doped PbO_2_ electrodes for efficient removal of sulfamethoxazole

**DOI:** 10.1038/s41598-024-58893-z

**Published:** 2024-04-05

**Authors:** Dancheng Zhu, Yifan Wu, Kai Zheng, Hao Xu, Chao Chen, Jun Qiao, Chao Shen

**Affiliations:** Key Laboratory of Pollution Exposure and Health Intervention of Zhejiang Province, College of Biology and Environmental Engineering, Zhejiang Shuren University, Hangzhou, 310015 China

**Keywords:** Electrocatalysis, Pollution remediation

## Abstract

In this study, we deposited Ti_3_C_2_T_x_-modified, rare-earth-doped PbO_2_ on the surface of a carbon fabric via electrodeposition. The surface morphology and electronic structure of the electrode were characterized with SEM, XRD and XPS. The layered Ti_3_C_2_T_x_ did not change the structure of β-PbO_2_, and at the same time, it improved the crystallinity of the material and reduced the grains of PbO_2_. Electrochemical experiments showed that the addition of Ti_3_C_2_T_x_ increased the electrochemical activity of the electrode and produced more H_2_O_2_, which contributed to the degradation of pollutants. The efficiency of sulfamethoxazole (SMX) degradation reached 95% after 120 min at pH 3 with a current density of 50 mA/cm^2^. Moreover, the electrode has good cycling performance, and the degradation efficiency was still 80% after 120 min after 10 cycles of recycling. Based on the intermediates identified by HPLC‒MS, a mechanism for SMX degradation was proposed. Our results will provide a new idea for the development of efficient electrocatalytic degradation of antibiotics.

## Introduction

Antibiotics are primarily used to treat diseases associated with infections in humans and animals^1–4^. Current treatment methods do not remove antibiotics effectively and pose significant risks to aquatic ecosystems and human health. Sulfonamides are common antibiotics used in aquatic environments^5^. However, the conventional activated sludge method does not degrade sulfonamide wastewater sufficiently, and the effluent quality generally fails to meet water quality standards^6^. Therefore, a water treatment technology that removes trace amounts of sulfamethoxazole (SMX) from wastewater is needed.

Electrochemical oxidation is a simple and effective method for the treatment of various organic pollutants in liquid media^7,8^. Rare earth doped PbO_2_ is a promising cathode material for electrochemical oxidations of organic pollutants. Doping with rare earth elements enabled Fenton-like reactions with H_2_O_2_ and Ce^4+^/Ce^3+^^9,10^ or Eu^3+^/Eu^2+^^11^. The rare earth elements increased the mass transfer rate of O_2_, such as Ce, which generally exists in the form of CeO_2_ particles. After the conversion of Ce^4+^ to Ce^3+^, the electrode generated oxygen vacancies and adsorbed O_2_ to generate H_2_O_2_. However, rare earth-modified PbO_2_ electrodes are generally hydrophobic and unstable^12^. In addition, hydrophobic electrodes have weak electron transfer capabilities for O_2_ reduction, which affect the efficiencies of H_2_O_2_ generation. This all affects the activities of rare-earth doped PbO_2_ electrodes, and further modification is needed for improved electrochemical activity.

Ti_3_C_2_T_x_, a new 2D material, was first discovered in 2011^13^. Compared with other two-dimensional materials, Ti_3_C_2_T_x_ has been widely used in Electrocatalytic applications due to its thin atomic layer, high electrical conductivity, high hydrophilicity, many active sites, and good mechanical properties.^14–19^. Ti_3_C_2_T_x_ increases the efficiencies of oxidation reactions and the electrochemical oxidation activity, which is essential for the production of H_2_O_2_^20,21^. Additionally, Ti_3_C_2_T_x_ increases the efficiency of H_2_O_2_ decomposition: (1) Ti^3+^ and Ti^2+^ lose electrons to form Ti^4+^ and produce oxidized material^22^; (2) Most of the functional groups are hydrophilic, which facilitates the activation of H_2_O_2_. Doping with Ti_3_C_2_T_x_ increases the conductivity of the electrode, thus reducing energy consumption^20^. In addition, the surface functional groups of 2D Ti_3_C_2_T_x_ adsorb organic pollutants through electrostatic interactions, hydrogen bonding, surface complexation, and π-π interactions^23,24^. This increases the rate of antibiotic diffusion. Therefore, Ti_3_C_2_T_x_ is an ideal material for electrode modification.

Therefore, we propose an innovative approach to fabricate an electrode material with a composite-structured electrode by compositing Ti_3_C_2_T_x_ with a rare-earth-modified PbO_2_ electrode. Based on an earlier report, we believe that by introducing Ti_3_C_2_T_x_, the ability of the PbO_2_ electrode to generate H_2_O_2_ can be effectively enhanced, thereby improving the electrode's activity for electrocatalytic degradation of antibiotics. In this study, we used carbon fabric (CF) as the substrate. The Eu-doped PbO_2_-CeO_2_-Ti_3_C_2_@CF anode material was prepared by electrochemical deposition. Ti_3_C_2_ was introduced into an electrochemical conservation system. The results showed that the concentration of hydrogen peroxide generated with the Ti_3_C_2_ anode was three times that of the anode without Ti_3_C_2_. Under certain conditions, the efficiency of sulfamethoxazole degradation reached 91% in 60 min, and the anode was recycled at least ten times.

## Methods

### Chemicals and materials

MAX (Ti_3_AlC_2_) powder (90%) was purchased from Xfnano Materials Tech Co., Ltd.; hydrofluoric acid (HF, 40%) and methanol (MeOH, 99.7%) was purchased from Sinopharm Chemical Reagent Co., Ltd.; dimethyl sulfoxide (DMSO, 99.7%), Pb(NO)_3_ (99%), and HNO_3_ (65–68%) were purchased from Lingfeng Chemical Reagent Co.; Eu(NO)_3_ (99.99%), CeO_2_ (99.95%), SMX (98%) was purchased from Shanghai Aladdin Biochemical Technology Co., Ltd.; and CF was purchased from Suzhou Zhengtairong New Material Co., Ltd.

### Preparation of Ti_3_C_2_T_x_

Multilayered Ti_3_C_2_ was prepared by HF etching^25^. Then, 200 mg of Ti_3_C_2_ was added to 20 mL of DMSO and stirred for 24 h at room temperature. Ti_3_C_2_ was separated by centrifugation and dried under vacuum at 90 °C for 24 h to obtain DMSO-intercalated Ti_3_C_2_. After drying, 50 mL of deionized water was added and sonicated for 2 h to obtain an aqueous Ti_3_C_2_T_x_ suspension.

### Preparation of Eu-doped PbO_2_-CeO_2_-Ti_3_C_2_@CF

The CF was cut into 2 × 2 cm pieces and cleaned by ultrasonication with nitric acid, ethanol and deionized water in turn for 15 min. First, 0.02 mol of Pb(NO_3_)_3_, 0.04 mol of Eu(NO_3_)_3_, 0.01 mol of HNO_3_ and 400 mg of CeO_2_ were added to 50 ml of the previously prepared Ti_3_C_2_T_x_ suspension, and deionized water was added to raise the total volume of the electrolyte 100 ml. The electrolyte was ultrasonically mixed for 30 min and then electrodeposited at a current intensity of 10 mA/cm^2^ for 40 min to prepare Eu-doped PbO_2_-CeO_2_-Ti_3_C_2_@CF. Different electrode materials were prepared by changing the amounts of Pb(NO_3_)_3_, Eu(NO_3_)_3_, CeO_2_ and Ti_3_C_2_T_x_ suspensions.

### Characterization

The surface structures of the electrodes were characterized with a Hitachi SU-70 scanning electron microscope (SEM), and the elemental distribution of the electrodes was characterized with a Bruker super-X energy dispersive X-ray spectrometer (EDS). X-ray diffraction (XRD) was performed with a Rigaku SmartLab SE diffractometer to analyze the crystal structures of the electrodes. X-ray photoelectron spectroscopy (XPS) was performed with a Thermo Scientific K-Alpha instrument and monochromatic Al Kα radiation to analyze the elemental composition, chemical state and molecular structure of the electrode surface.

Cyclic voltammetry (CV) and linear scanning voltammetry (LSV) were performed with an electrochemical workstation and a three-electrode system (CHI 760E, Shanghai Chenhua Instruments Co., Ltd., China). Eu-PbO_2_-CeO_2_@CF or Eu-PbO_2_-CeO_2_-Ti_3_C_2_@CF were used as the working electrodes, Pt sheets as the counter electrodes, and saturated mercuric glycol electrodes as the reference electrodes. The electrode was tested in a 0.05 M sodium sulfate solution at pH 3. CV was performed in the voltage range − 0.8 to 1 V with a sweep rate of 10 mV/s. LSV was performed in the voltage range − 1.2 to 0 V with a sweep rate of 10 mV/s.

### Electrolysis

Electrolysis was conducted with a galvanostat to investigate the electrochemical performance of Eu-PbO_2_-CeO_2_-Ti_3_C_2_@CF. The antibiotics were electrolyzed in a 100 mL electrolytic cell with 0.05 M Na_2_SO_4_, the electrode prepared above served as the cathode and a platinum sheet as the anode, and the solution was electrolyzed in a 100 mL electrolytic cell with 0.05 Na_2_SO_4_ used as the electrolyte, the prepared electrode was used as the cathode, and the Pt sheet as the anode. A magnetic stirrer was used to stir the solution during the electrolysis. The effects of different experimental conditions (initial concentration, pH, and current intensity) on the degradation of SMX were explored. After every 20 min, a 3 ml water sample was taken, the SMX solution was scanned with an ultraviolet and visible (UV‒Vis) spectrophotometer (Thermo Fisher Evolution 201) over the wavelength range 200–450 nm, and the concentration of SMX was determined from the peak at 262 nm. The concentration of H_2_O_2_ was analyzed by the potassium oxalate titanium method. Analyzing TOC in electrolytes by Shimadzu TOV-V CPH. Pb ion concentrations were measured by inductively coupled plasma (ICP, Agilent 720ES).

### High-performance liquid chromatography‒mass spectrometry (HPLC‒MS)

Under the optimal reaction conditions, the solutions reacted for different periods were taken to determine the intermediate products. The method used an Agilent 1290-6465Q-TOF HPLC‒MS system for the analyses. A C18 chromatographic column was used with a column temperature of 40 °C, a mobile phase of 40% acetonitrile and 60% pure water, including 1% HPLC grade acetic acid, a flow rate of 1 mL/min and a detection wavelength of 269 nm with an injection volume of 20 μL, and a full-scan acquisition. The mass spectra of the compounds were acquired in positive ion mode for m/z between 100 and 400.

## Results and discussion

**Figure 1:**
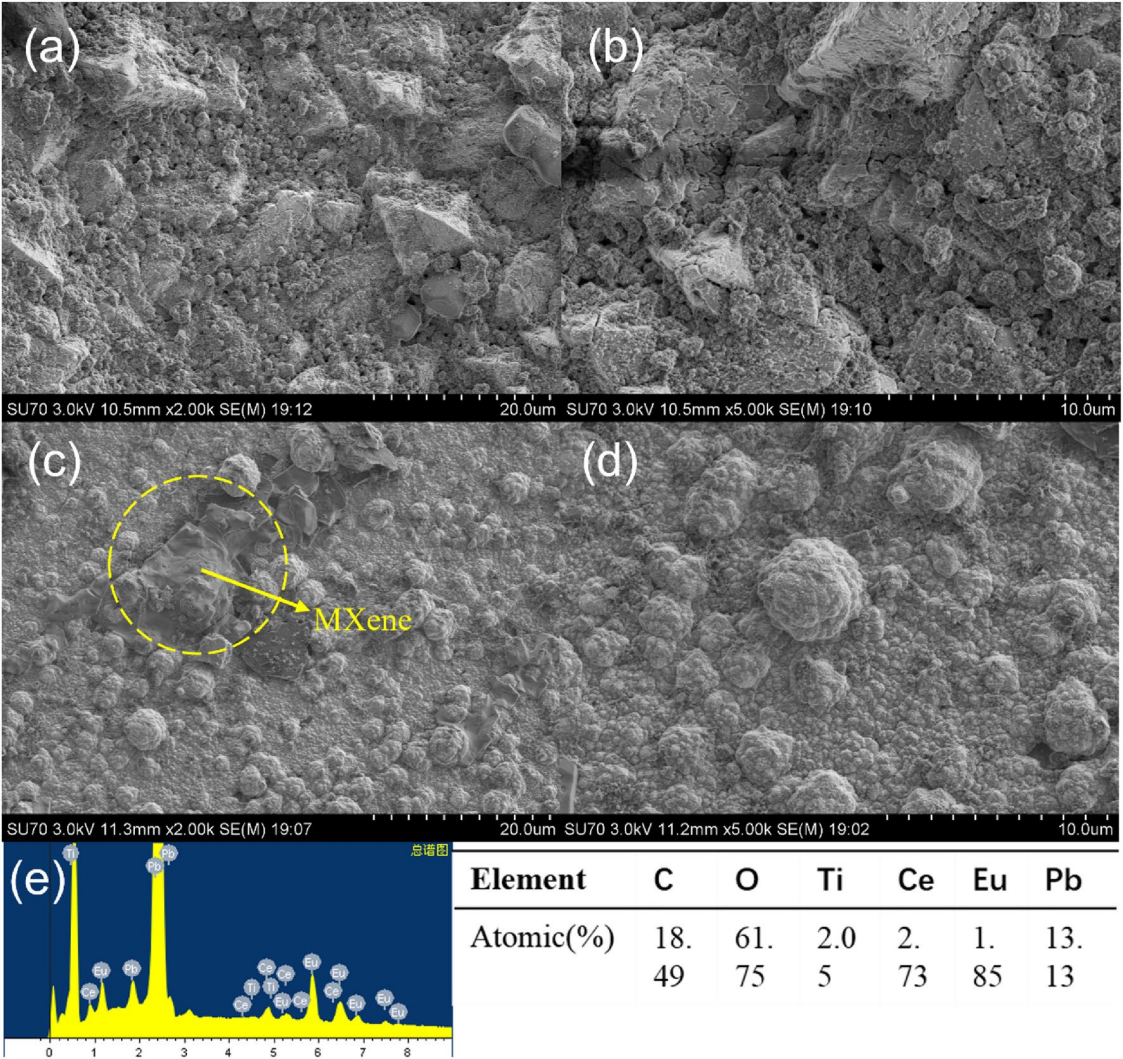
SEM images of (**a**,**b**) Eu-doped PbO_2_-CeO_2_@CF and (**c**,**d**) Eu-doped PbO_2_-CeO_2_-Ti_3_C_2_@CF, (**e**) EDS spectra of Eu-doped PbO_2_-CeO_2_-Ti_3_C_2_@CF.

The surface morphologies of the Eu-PbO_2_-CeO_2_@CF (Fig. 1a,b) and Eu-PbO_2_-CeO_2_-Ti_3_C_2_@CF (Fig. 1c,d) electrodes were characterized with SEM. The Eu-PbO_2_-CeO_2_@CF particles were mainly pyramidal with rough surfaces and obvious height differences. With the introduction of Ti_3_C_2_, the grain sizes decreased obviously, and the surface was smoother and contained some circular polygonal nanocrystals. In addition, there were sheet-like structures on the surface of the Eu-PbO_2_-CeO_2_-Ti_3_C_2_@CF electrode, which were speculated to be Ti_3_C_2_ nanosheets. Compared to PbO_2_ electrodes (Fig. S1a), this electrode with a complex surface morphology and small crystal sizes had a large electrochemically active surface area. The compositions of the Eu-PbO_2_-CeO_2_@CF (Table S1) and Eu-PbO_2_-CeO_2_-Ti_3_C_2_@CF (Fig. 1e) electrodes were characterized with EDS, and the Eu-PbO_2_-CeO_2_@CF electrode was composed of Eu, Pb, Ce, O and C, confirming the presence of Eu and CeO_2_. The EDS analysis of Eu-PbO_2_-CeO_2_-Ti_3_C_2_@CF showed that, in addition to the above 5 elements, Ti was also observed, and the content was 2.05 at.%, which also confirmed the presence of Ti_3_C_2_.

**Figure 2:**
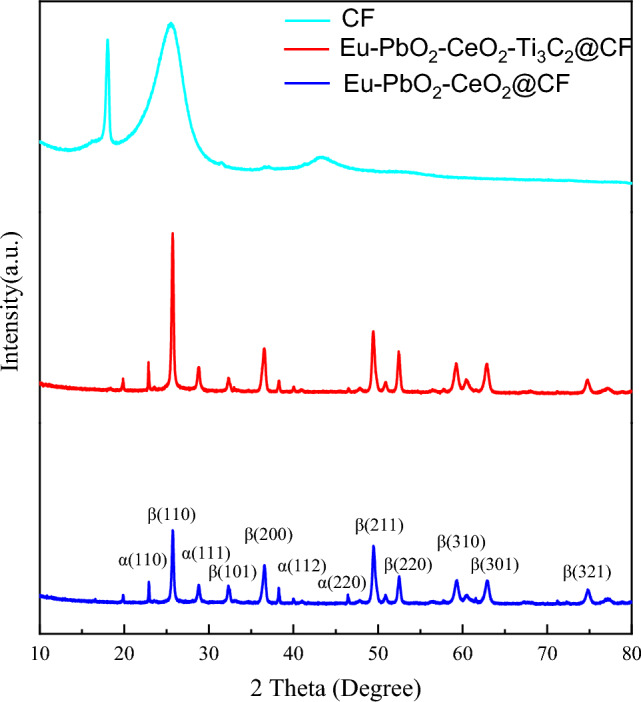
XRD spectra of CF, Eu-doped PbO_2_-CeO2@CF and Eu-doped PbO_2_-CeO_2_-Ti_3_C_2_@CF.

The structures of the CF, Eu-PbO_2_-CeO_2_-Ti_3_C_2_@CF and Eu-PbO_2_-CeO_2_-Ti_3_C_2_@CF electrode were explored with XRD (Fig. 2). As seen from the graphs, the high intensities and narrow shapes of the diffraction peaks for both electrodes indicated that the electrodes were highly crystalline, suggesting that the modification with Ti_3_C_2_ did not affect the cleanliness of the PbO_2_ substrate. Additionally, the XRD diffraction peaks were compared with those on standard PDF cards (α-PbO_2_ and β-PbO_2_). Most of the diffraction peaks for the two electrodes matched the diffraction peaks of β-PbO_2_, and a few matched the diffraction peaks of α-PbO_2_, indicating that the main component of the electrodes prepared by this method was β-PbO_2_. The UV–Vis spectra (Fig. S2) also confirmed that the introduction of Ti_3_C_2_ did not change the structure of the electrode. In addition, based on the XRD spectra (Fig. S1b), the proportion of β-PbO_2_ in the Eu-PbO_2_-CeO_2_@CF electrode was much larger than that in PbO_2_-CeO_2_@CF. α-PbO_2_ had a higher electrochemical activity and lower stability than β-PbO_2_. The grain sizes of both were calculated separately by Scheller's formula, and the grain size of added Ti_3_C_2_ was 10.5 nm, which was much smaller than the size (28.5 nm) of unadded Ti_3_C_2_. The previous SEM image (Fig. 1a) also showed that the surface structure of Eu-PbO_2_-CeO_2_@CF was less dense and easily peeled off during the electrochemical process. In contrast, after the addition of Ti_3_C_2_T_x_, the basic structure was still β-PbO_2_, but the surface morphology was denser (Fig. 1c) with better stability and less likely to be peeled off.

**Figure 3:**
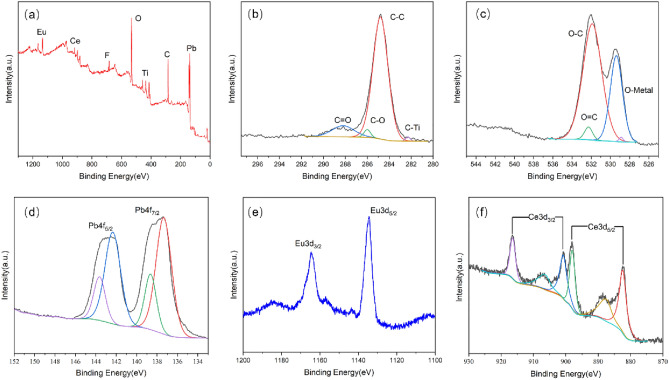
XPS spectra of Eu-doped PbO_2_-CeO_2_-Ti_3_C_2_@CF; (**a**) survey spectrum and (**b**) C 1s, (**c**) O 1s, (**d**) Pb 4f, (**e**) Eu 3d and (**f**) Ce 3d high resolution spectra.

The chemical and electronic states of Eu-PbO_2_-CeO_2_-Ti_3_C_2_@CF were analyzed by XPS (Fig. 3). During preparation of the Ti_3_C_2_ nanosheets, many functional groups were generated on the surface, such as OH^–^, O^2–^, and F^–13^. Their presence was verified by XPS (Fig. 3a). Figure 3b shows the C 1s spectrum of Eu-PbO_2_-CeO_2_-Ti_3_C_2_@CF. C-Ti bonds were present in the samples, which indicated that the Ti_3_C_2_ nanosheets retained their original properties after electrodeposition. The XPS spectrum of the Eu-PbO_2_-CeO_2_@CF electrode did not show the same peaks (Fig. S3a). Figure 3c shows the XPS spectrum of the O 1s core layer. The O 1s spectrum had three peaks at 528.8 eV, 531.9 eV and 532.3 eV associated with the Pb–O, surface O-C and O = C groups, respectively. The typical Pb 4f XPS peaks (Fig. 3d) at 137.6 eV for Eu-PbO_2_-CeO_2_-Ti_3_C_2_@CF were assigned to Pb^4+^. The peak at 138.5 eV is the formation of a small amount of PbCO_3_ on the surface as a result of the binding of PbO_2_ to CO_2_ in the air. Figure 3e,f shows the Eu 3d and Ce 3d XRD data. Ce 3d has a clear spin–orbit splitting peak and the Ce 3d_5/2_ peak at the rightmost 882.18 eV confirms the presence of CeO_2_ nanoparticles.

**Figure 4:**
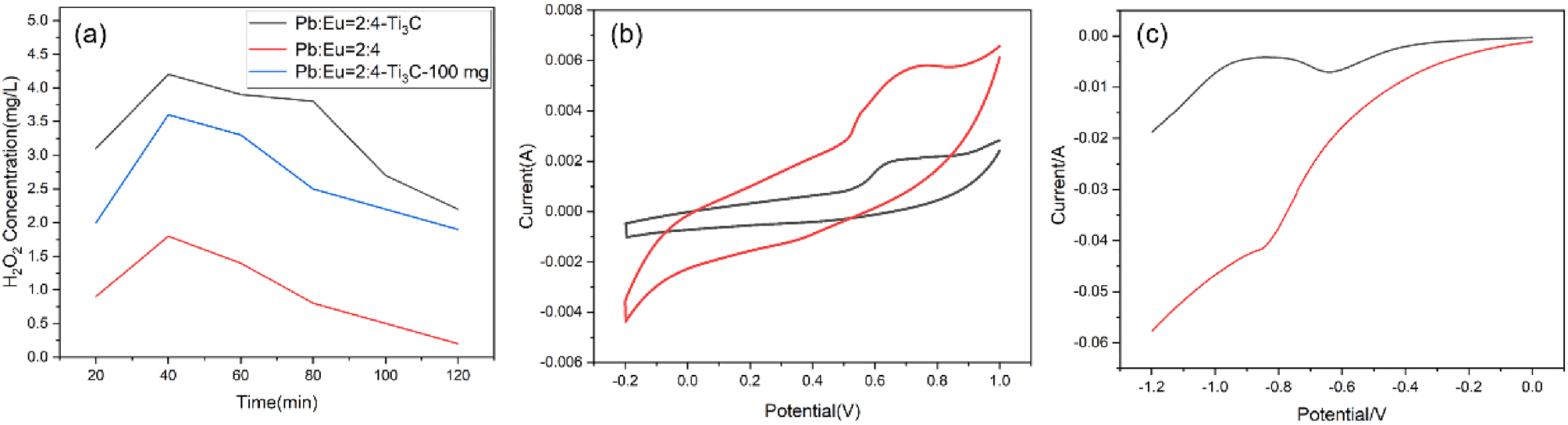
(**a**) Evolution of the H_2_O_2_ concentration, (**b**) CV data, and (**c**) LSV data for Eu-doped PbO_2_-CeO_2_@CF (black) and Eu-doped PbO_2_-CeO_2_-Ti_3_C_2_@CF (red).

The H_2_O_2_ generated in the electrochemical oxidation system is critical for the degradation of SMX. Therefore, the concentration of H_2_O_2_ was studied spectrophotometrically with potassium titanyl oxalate (Fig. 4a). The concentration of H_2_O_2_ increased sharply in the first 20 min, reaching a peak at 40 min. The activity of the electrode in generating H_2_O_2_ increased with the addition of Ti_3_C_2_. The electrode prepared by adding 200 mg of Ti_3_C_2_ to the electrolyte produced three times more H_2_O_2_ than the electrode without Ti_3_C_2_ and 1.5 times more H_2_O_2_ than the electrode with 100 mg of Ti_3_C_2_. The CV and LSV curves of the Eu-PbO_2_-CeO_2_@CF (black) and Eu-PbO_2_-CeO_2_-Ti_3_C_2_@CF (red) electrodes were then generated separately. As shown in Fig. 4b, the redox peak current and peak area for Eu-PbO_2_-CeO_2_-Ti_3_C_2_@CF were significantly greater than those for Eu-PbO_2_-CeO_2_@CF. Figure 4c shows that the PbO_2_-CeO_2_-Ti_3_C_2_@CF cathode had a stronger current response when a strong reduction peak corresponding to the ORR process was observed, as the Ti_3_C_2_ material significantly improved the electron transfer and catalytic activity of the ORR. Thus, when assembled into an electrochemical oxidation system, the PbO_2_-CeO_2_-Ti_3_C_2_@CF cathodes enabled in situ generation of -OH and contaminant degradation.

To determine the optimal ratio of Pb(NO_3_)_3_, Eu(NO_3_)_3_ and CeO_2_, the efficiencies of SMX degradation at these electrodes were studied by varying the concentrations in the electrolyte and under established conditions (an initial SMX concentration of 30 mg/L, an initial pH of 3 and an applied current density of 50 mA/cm^2^) (Fig. S4). The electrolytic efficiencies of the prepared electrodes were highest when the concentrations of Pb(NO_3_)_3_, Eu(NO_3_)_3_ and CeO_2_ were 2 mol/L, 4 mol/L and 2 g/L, respectively. The rate of SMX degradation reached 85% after 2 h.

**Figure 5:**
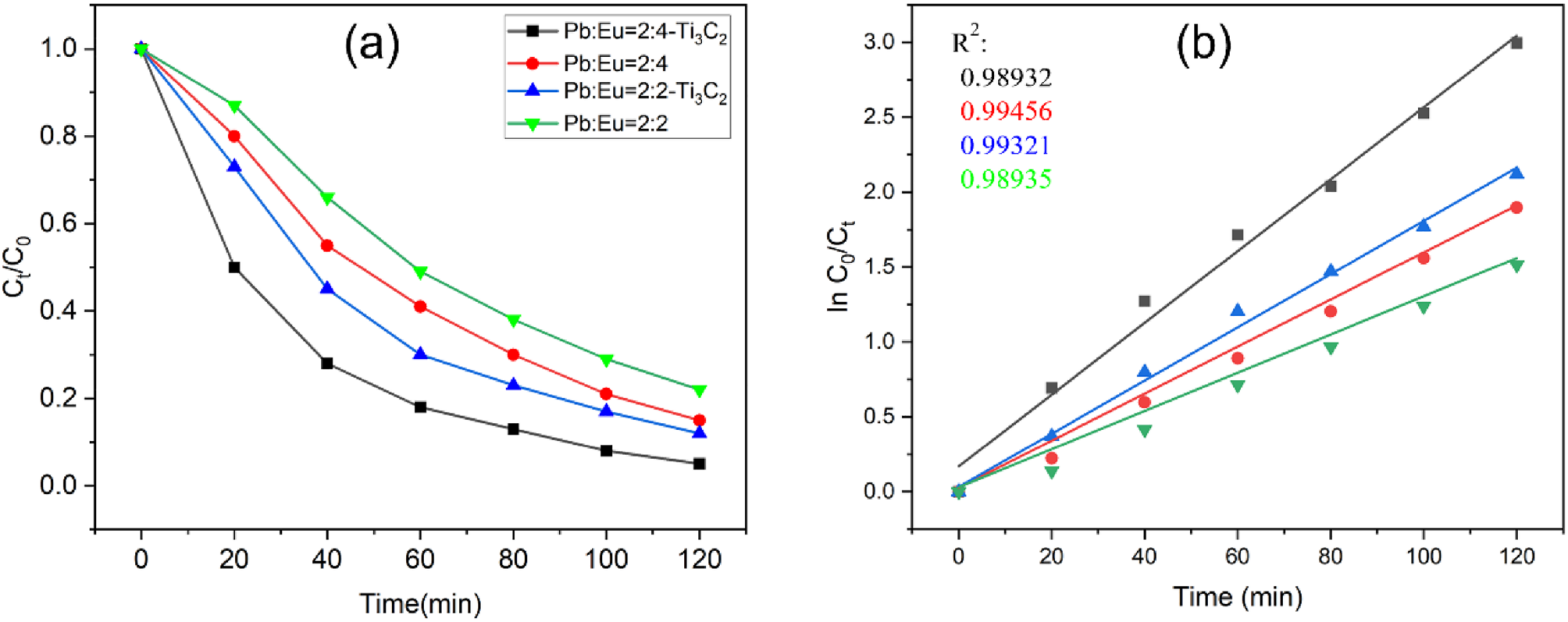
(**a**) SMX degradation with Eu-doped PbO_2_-CeO_2_@CF and Eu-doped PbO_2_-CeO_2_-Ti_3_C_2_@CF and (**b**) kinetic analyses with the pseudo-first-order model.

Based on these results, 2 g/L Ti_3_C_2_T_x_ was added to the electrolyte, and the efficiency of SMX degradation was investigated under the same conditions (Fig. 5a). For electrodes with different Pb and Eu ratios, the addition of Ti_3_C_2_T_x_ improved the rate of SMX degradation. For the electrode with a Pb: Eu ratio of 2:4, the degradation efficiency increased by 10% with the addition of Ti_3_C_2_T_x_ and reached 95% at 120 min. Then the TOC in the electrolyte was detected by a TOC analyzer, and it was 4.04 mg/L, with a removal rate of 71.6%. According to the relevant literature^26–29^, the concentration of SMX currently has been much smaller than the minimum inhibitory concentration. During degradation, the rate of the electrode reaction was calculated as:

1 $${\text{ln C}}_{0} /{\text{C}}_{{\text{t}}} = {\text{ kt}}$$

where C_0_ (mg/L) and C_t_ (mg/L) are the SMT concentrations at time 0 and t min, respectively (Fig. 5b). All four electrodes fit the first order kinetic model. With the addition of Ti_3_C_2_T_x_, the electrolytic (Pb:Eu = 2:4) degradation rate constantly increased from 0.0157 ± 0.00052 to 0.0240 ± 0.0011. Moreover, the degradation rate of the electrode without Ti_3_C_2_T_x_ was slower in the first 20 min compared to that of the electrode with Ti_3_C_2_T_x_. This was mainly due to the reduction rate of O_2_ on the hydrophobic surface and the lower efficiency of H_2_O_2_ activity. The addition of Ti_3_C_2_T_x_ increased the hydrophilic functional groups and improved the degradation activity. The electrode degradation activity of Eu-PbO_2_-CeO_2_-Ti_3_C_2_@CF was compared with that of electrodes reported in the literature (Table 1). Eu-PbO_2_-CeO_2_-Ti_3_C_2_@CF showed excellent activity at higher initial concentrations of SMX and without additional ventilation.

**Table 1:** Comparison of Eu-doped PbO_2_-CeO_2_-Ti_3_C_2_@CF with some recently reported electrodes for SMX degradation.

Entry	Electrodes	Degradation Condition	Removal efficiency (%)	References
1	Eu-doped PbO_2_-CeO_2_-Ti_3_C_2_@CF	30 mg/L, pH: 3 50 mA/cm^2^	95	This work
2	Mn_0.67_Fe_0.33_-MOF-74@CF	10 mg/L, pH: 3 30 mA/ cm^2^ Continuously ventilated	96	Ref.^2^
3	Nb/BDD	10 mg/L 2.5 A Oxygen injection	95	Ref.^5^
4	GAC@Ni/Fe	1 mg/L 5 V Continuously ventilated	90.8	Ref.^43^

**Figure 6:**
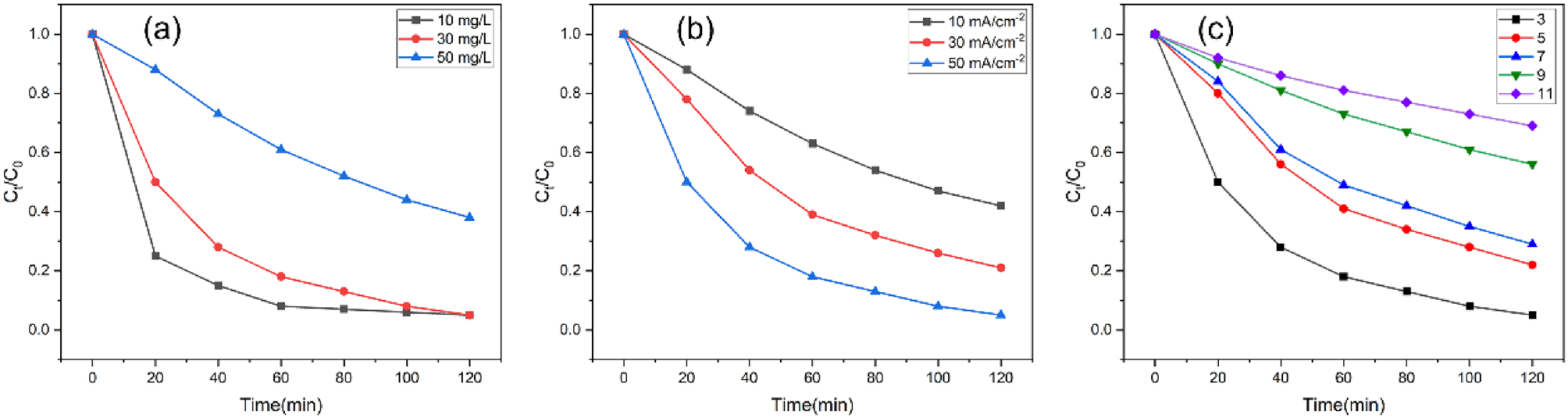
Effects of (**a**) SMX initial concentration, (**b**) current density, and (**c**) initial pH of the solution on the removal of SMX by the Eu-doped PbO_2_-CeO_2_-Ti_3_C_2_@CF electrode.

To demonstrate the high electrocatalytic activity of the electrode, the effects of different operating parameters (including the initial concentration, current density and pH) on the electrochemical oxidation efficiency were investigated (Fig. 6).

Figure 6a shows the effect of initial SMX concentrations on SMX degradation efficiency. The experimental conditions included a current density of 50 mA/cm^2^, a pH of 3 and SMX concentrations of 10, 30 and 50 mg/L. As the concentration of the pollutant decreased, the degradation efficiency of SMX increased, but the amount of degradation decreased. This was mainly because at low concentrations, electrocatalytic oxidation was faster than diffusion, thus allowing effective degradation of the organic matter. As the concentration of the pollutant increased, so did the amount of organic matter produced during degradation, including pollutants and intermediate products. In addition, the electrode produced a limited number of hydroxyl radicals. As the pollutant concentration increased, the number of hydroxyl radicals acting per unit of pollutant decreased, which made the degradation of SMX less effective^30^. Therefore, an SMX concentration of 30 mg/L was chosen for subsequent experiments.

The current density is a key factor in the electrochemical oxidation process because it regulates the generation of hydroxyl radicals^31^. Figure 6b shows the rate of SMX removal for different applied current densities. As the current density was increased from 10 to 50 mA/cm^2^, the efficiency of SMX removal increased from 58 to 95%. When the current density was increased from 30 to 50 mA/cm^2^, the increase in the degradation efficiency was smaller than before. The higher current density may have enhanced the reaction of oxygen on the anode surface, thus competing with the oxidation of organic matter on the electrodes surface and affecting the removal efficiency^32^. In addition, the diffusion rate of contaminants to the electrode is limited at the same concentration, limiting the degradation rate at high currents and decreasing the current efficiency. Therefore, after careful consideration, the SMX degradations were performed at a current density of 50 mA/cm^2^.

Figure 6c shows the effect of different pHs on the efficiency of SMX degradation^33^. The results show that the fastest SMX degradation rate was achieved at pH 3. This is because hydroxyl radicals are more favorable for SMX degradation under acidic conditions^34^. In addition, CO_2_ was the main product from electrochemical degradation of SMX, and under alkaline conditions, CO_2_ dissolved in the solution and inhibited pollutant oxidation. Based on the above analysis, pH 3 was used as the initial pH for the electrochemical degradation of SMX.

**Figure 7:**
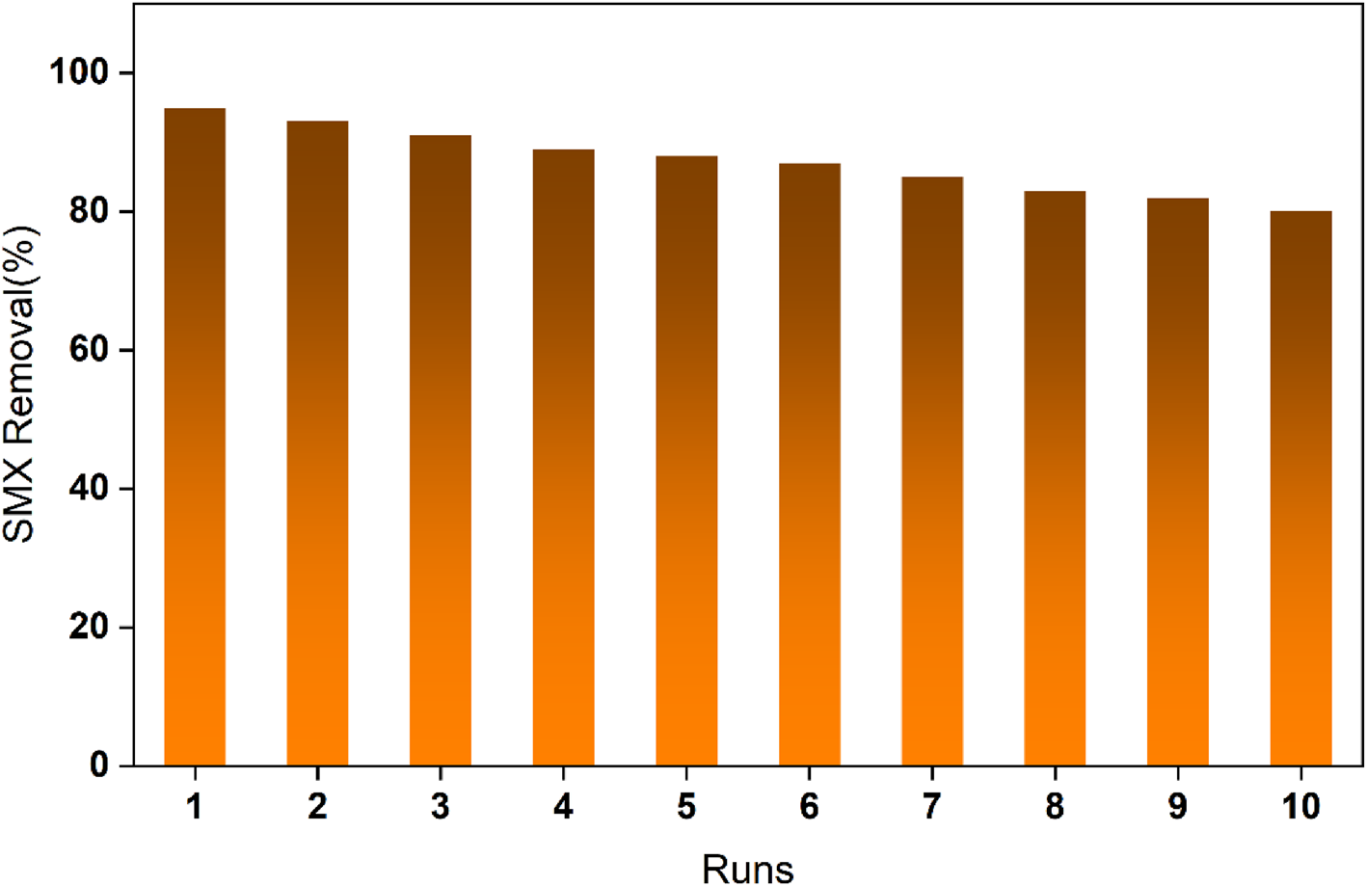
Recycling and reuse of the Eu-doped PbO_2_-CeO_2_-Ti_3_C_2_@CF electrode.

The cycling performance of the electrodes was investigated. The SMX cycled for 120 min under the optimal conditions for SMX degradation. After each run, the degradation rate of SMX decreased slightly and remained at 80% after 10 cycles (Fig. 7). In addition, the electrodes after electrochemical degradation were characterized by XRD (Fig. S5), it can be found that the structure as well as the morphology did not change significantly. In addition, the content of Pb ions in the electrolyte was detected by ICP-MS, and the concentration of Pb ion released from the Eu-PbO_2_-CeO_2_-Ti_3_C_2_@CF electrode was 3.68 μg/L, which is less than the World Health Organization's requirements for lead ion concentrations in drinking water and China's permitted effluent discharge standards for Pb in surface waters. Therefore, the electrochemical degradation of this electrode is safe and reliable for the environment and human health. Therefore, the Eu-PbO_2_-CeO_2_-Ti_3_C_2_@CF electrode had good stability.

To further investigate the mechanism of SMX degradation, methanol was used as a scavenger (Fig. S6) to investigate the role of ·OH. It can be found that the degradation efficiency of SMX decreased significantly after the addition of methanol, indicating that ·OH plays a major role in the degradation process.

**Figure 8:**
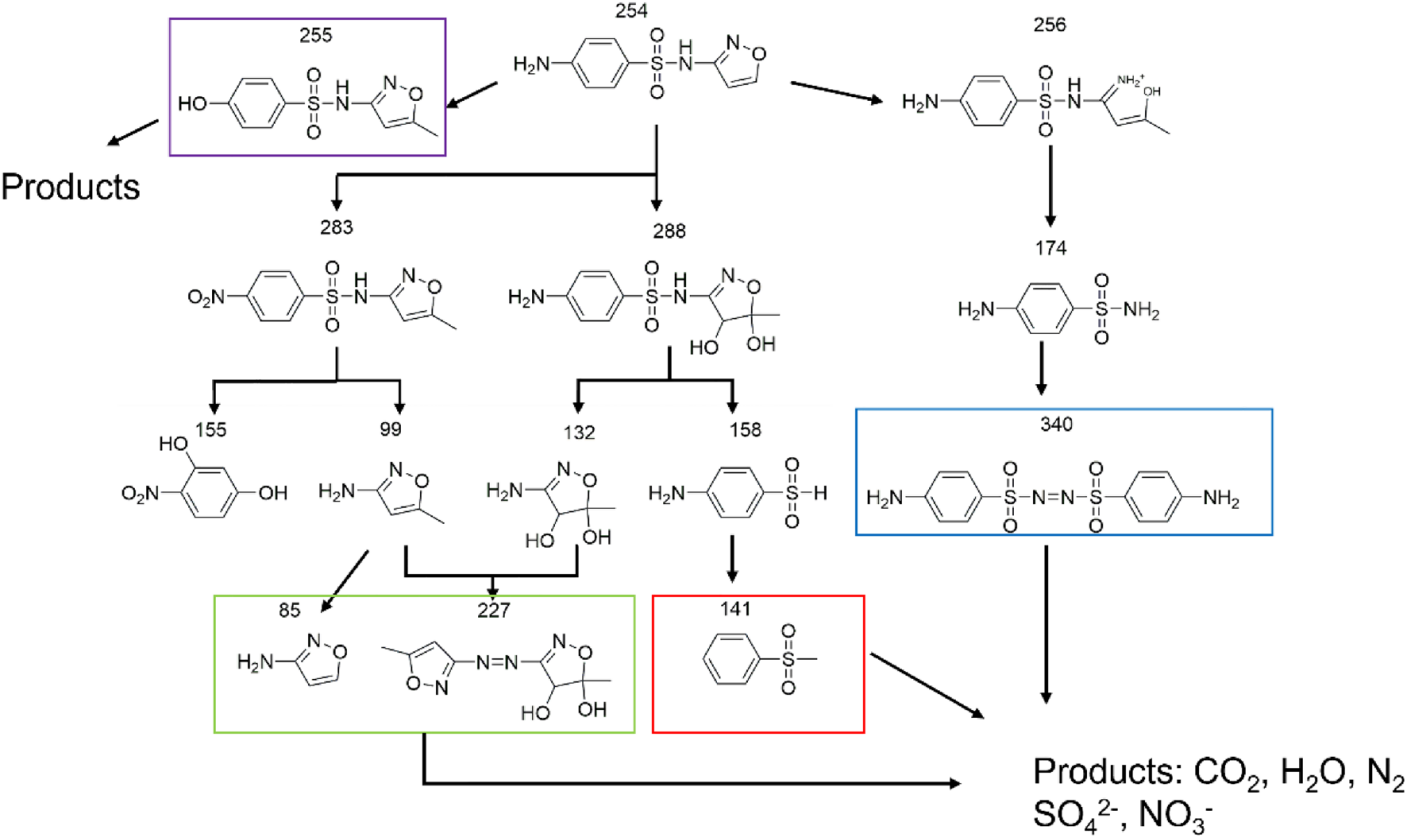
Degradation pathways for SMX.

To further investigate the degradation mechanism, The intermediates produced during SMX degradation were characterized by HPLC–MS (Table S2). Possible degradation pathways have been proposed in conjunction with the literature (Fig. 8).

In pathway I, the degradation pathway was hydroxylation of the arene ring. The aniline portion was attacked by the hydroxyl radical, resulting in the disappearance of the amine group and the formation of 255^35^.

In pathway II, the amino group on the arene ring in SMX was attacked by a hydroxyl radical to form NO_2_-SMX (283)^36^. Subsequently, the S‒N bond of NO_2_-SMX was cleaved to give 155 and 99^37^. In addition, ·OH react with the isoxazole ring to form 288^38^. Subsequently, the C-N bond breaks and 158 and 132 are formed. Then, 158 lost a -NH_2_ to form 141. Ions 132 and 99 coupled with N-centered radicals to form 227^39^. The remaining 99 was stripped of a methyl group to form 85^40^.

In pathway 3, the isoxazole ring in SMX opened to form 256, followed by C-N bond breakage to form 174^41^. Subsequent coupling was centered around the N atom to form 340^42^. All intermediates eventually degrade to water, carbon dioxide and inorganic ions.

## Conclusion

In summary, Ti_3_C_2_T_x_-modified rare earth element-doped PbO_2_ electrodes were prepared via electrodeposition and were fully characterized by SEM, XRD, and XPS. The results showed that Ti_3_C_2_T_x_ was doped into the PbO_2_ electrode and optimized the surface morphology as well as the electronic structure of the electrode. The optimal degradation conditions for the electrochemical degradation of SMX by electrodes were also investigated. The electrodes showed good stability and were recycled and reused at least 10 times. In addition, a degradation pathway was proposed based on an analysis of the HPLC–MS intermediates. All these results indicate that Ti_3_C_2_T_x_ is an ideal electrochemical oxidation modification material, which can effectively improve the electrochemical activity of the electrode. This work provides a new idea for electrode modification, which has a broad and great application prospect in treating difficult-to-degrade medical wastewater.

### Supplementary Information

Supplementary Information.

## Data Availability

The datasets used and/or analyzed during the current study available from the corresponding author on reasonable request.
