# Supplementary material for: Preparation of Ti3C2Tx modified rare earth doped PbO2 electrodes for efficient removal of sulfamethoxazole
Source: Sci Rep. 2024 Apr 5;14:8068. doi: 10.1038/s41598-024-58893-z (PMC10997634; doi:10.1038/s41598-024-58893-z)
Supplement: Supplementary file 1 — Supplementary Information. [file 41598_2024_58893_MOESM1_ESM.docx]

Supplementary material for

**Preparation of Ti_3_C_2_T_x_ Modified Rare Earth Doped PbO_2_ Electrodes for the Efficient Removal of SMX.**

Dancheng Zhu^1^, Yifan Wu^1^, Kai Zheng^1^, Hao Xu^1^, Chao Chen^1^, Jun Qiao^1^, Chao Shen^1a^

^1^Key Laboratory of Pollution Exposure and Health Intervention of Zhejiang Province, College of Biology and Environmental Engineering, Zhejiang Shuren University, Hangzhou 310015, China. E-mail: shenchaozju@163.com


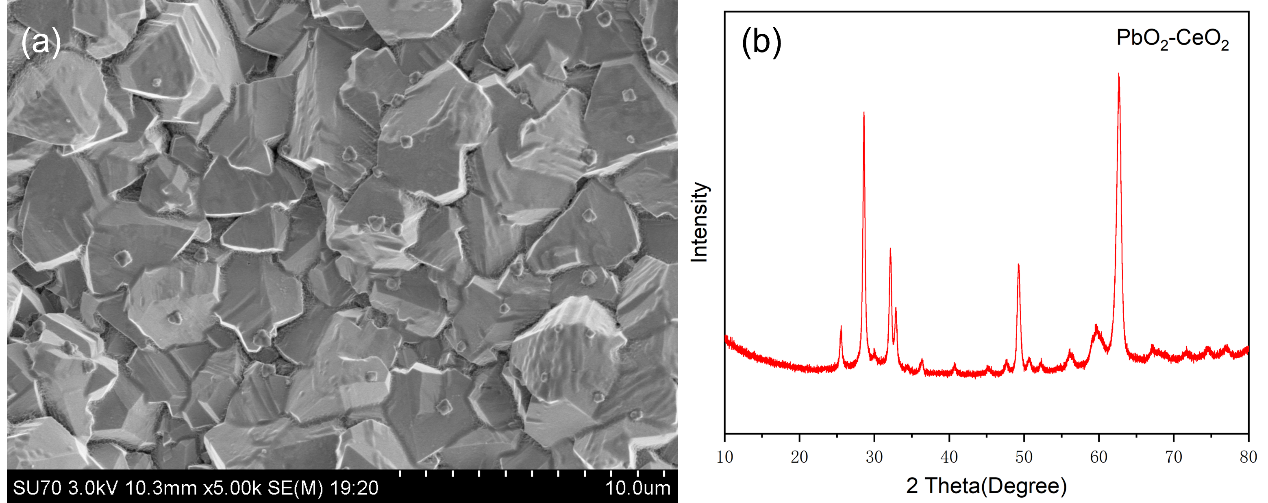


**Figure S2.** (a)SEM image of PbO_2_ @CF, (b) XRD spectra of PbO_2_-CeO_2_@CF

**Table S1.** EDS of Eu-doped PbO_2_-CeO_2_@CF

| Element | Eu-PbO_2_-CeO_2_@CF Atomic(%) |
| --- | --- |
| C | 21.54 |
| O | 56.92 |
| Ti | none |
| Ce | 3.90 |
| Eu | 3.75 |
| Pb | 13.88 |

**Figure S1.** UV-Vis analysis absorbance spectra of Eu-doped PbO_2_-CeO_2_-Ti_3_C_2_@CF and Eu-doped PbO_2_-CeO_2_@CF.

**
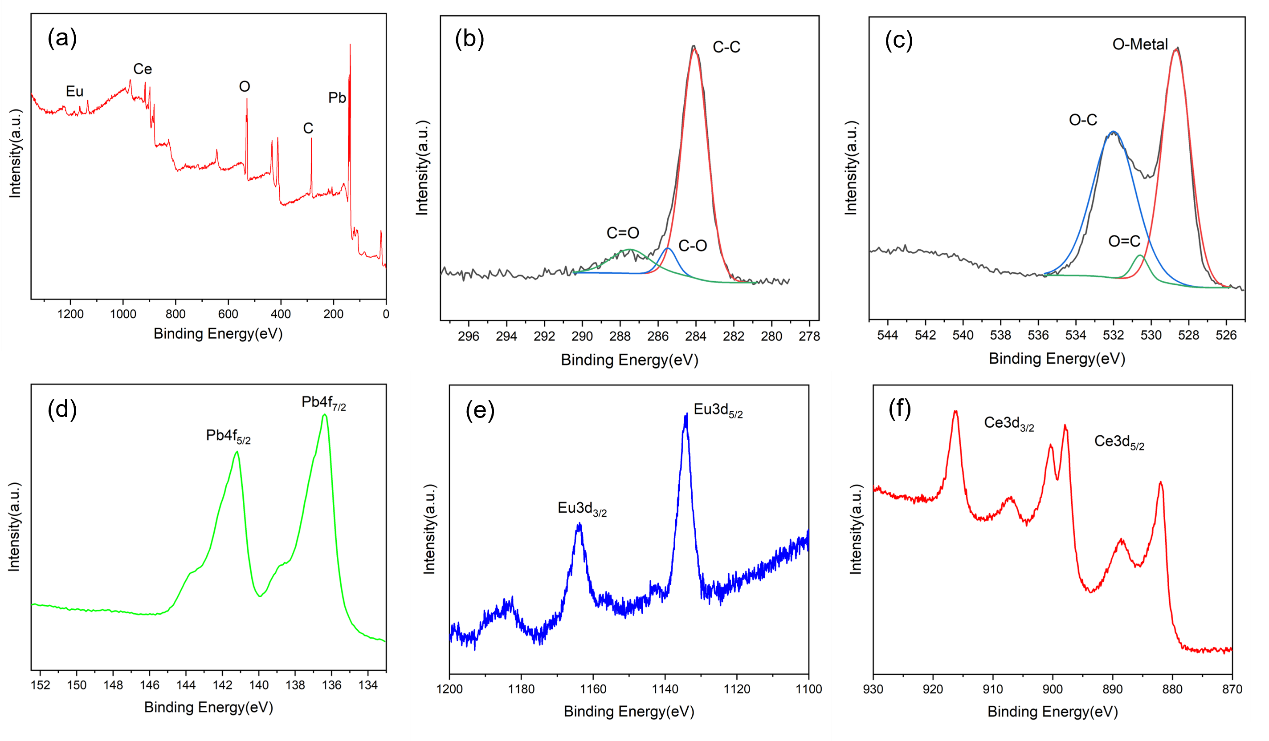
**

**Figure S3.** XPS spectra of Eu-doped PbO_2_-CeO_2_ @CF (a) wide, (b) C_1s_, (c) O_1S_, (d) Pb_4f_, (e) Eu_3d_, (f) Ce_3d_,


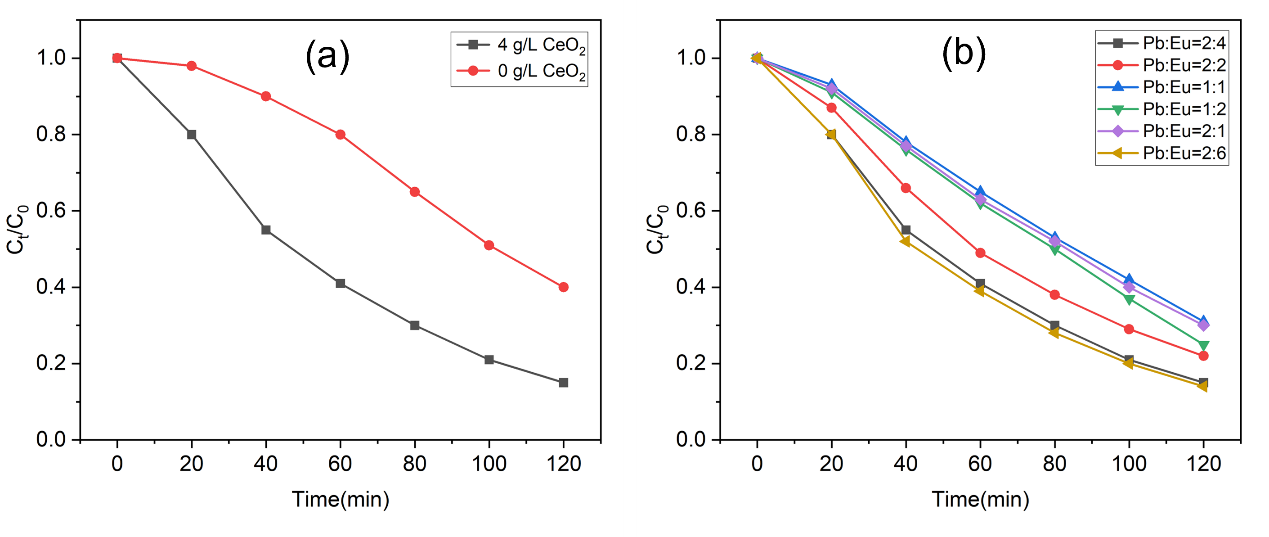


**Figure S4.** SMX degradation with different ratios of electrodes


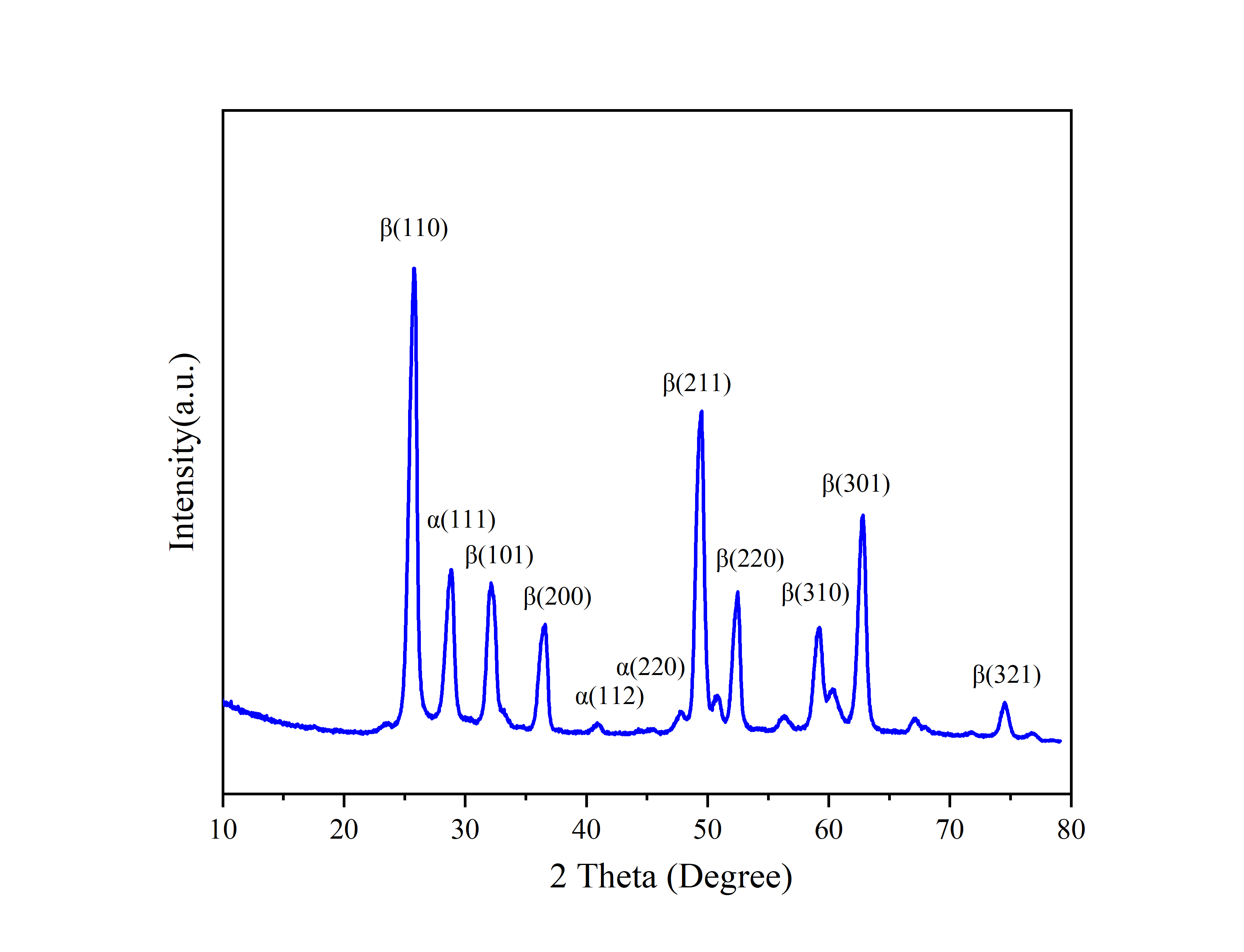


**Figure S5.** XRD spectra of Eu-doped PbO_2_-CeO_2_ -Ti_3_C_2_@CF after electrochemical degradation of antibiotics


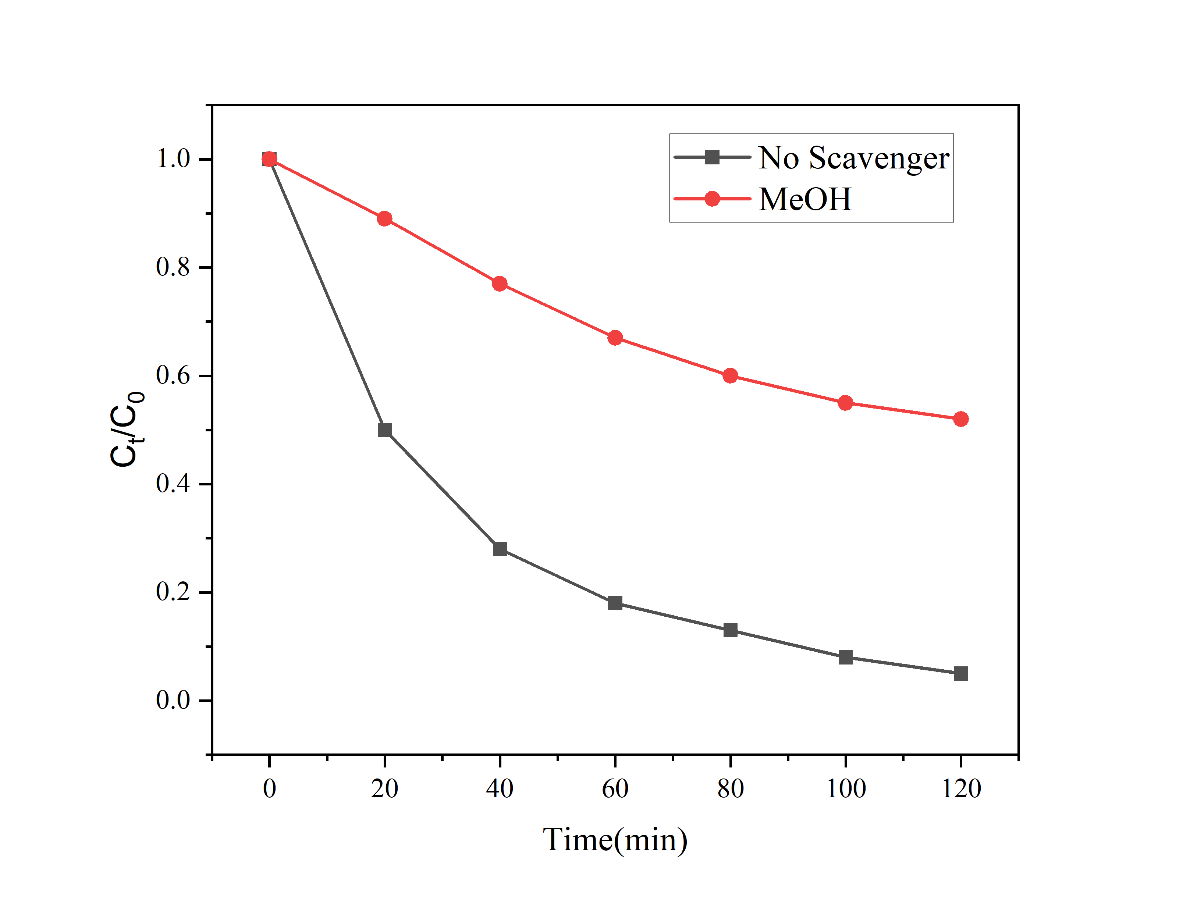


**Figure S6.** Degradation of SMX in the presence of MeOH.

**Table S2.** Information on the possible presence of intermediates.

| **m/z** | **Structured** |
| --- | --- |
| 254 |  |
| 255 |  |
| 283 |  |
| 288 |  |
| 155 |  |
| 99 |  |
| 132 |  |
| 158 |  |
| 141 |  |
| 227 |  |
| 85 |  |
| 174 |  |
| 340 |  |
